# The Effect of Probiotics on Health in Pregnancy and Infants: A Randomized, Double-Blind, Placebo-Controlled Trial

**DOI:** 10.3390/nu17111825

**Published:** 2025-05-28

**Authors:** Sylvie Binda, Mélanie Chow-Shi-Yée, Saly El Salti, Noémie Auclair-Ouellet, Marie-Laure Oula, Thomas Carton, Sébastien Leuillet, Diego Tomassi, Robert Hemmings, Isaac-Jacques Kadoch

**Affiliations:** 1 Rosell Institute for Microbiome and Probiotics, Montreal, QC H4P 2R2, Canada; selsalti@lallemand.com (S.E.S.); nauclairouellet@lallemand.com (N.A.-O.); moula@lallemand.com (M.-L.O.); 2 Clinique Ovo, Montreal, QC H4P 2S4, Canada; m.csy@cliniqueovo.com (M.C.-S.-Y.); r.hemmings@cliniqueovo.com (R.H.); j.kadoch@cliniqueovo.com (I.-J.K.); 3 Biofortis, 44800 Saint-Herblain, France; thomas.carton@biofortis.fr (T.C.); sebastien.leuillet@biofortis.fr (S.L.); diego.tomassi@biofortis.fr (D.T.); 4 Faculty of Medicine and Health Sciences, Department of Obstetrics-Gynecology, McGill University, Montreal, QC H4A 3J1, Canada; 5 Faculty of Medicine, Department of Obstetrics-Gynecology, Université de Montréal, Montreal, QC H3C 3J7, Canada

**Keywords:** probiotics, pregnant women, infants, *Lacticaseibacillus rhamnosus* Rosell^®^-11, *Bifidobacterium bifidum* HA-132, microbiome, microbiome establishment, vertical transfer

## Abstract

**Background/Objectives:** There is growing interest in the benefits of probiotic supplementation during pregnancy and lactation, but evidence supporting the beneficial effects for mother–infant dyads remains scarce. This study assessed the effects of probiotic supplementation on infection frequency and immunity in pregnant women and infants, and on microbiome establishment during the first month of life. **Methods:** At 28 weeks of gestation, 180 healthy pregnant women were randomized to receive either a placebo (*n* = 90) or a probiotic supplement (*n* = 90), Prenatis™, containing 5 billion CFU/day of *Lacticaseibacillus rhamnosus* Rosell^®^-11 and *Bifidobacterium bifidum* HA-132. **Results:** There was a significantly lower number of women with one or more infections during the study in the probiotics group (8 vs. 18, *p* = 0.05) and a trend for a lower number of infections during pregnancy (primary outcome) in the probiotics group (*p* = 0.07). Regarding infants, a lower number of days with infections during the first month of life was observed in the probiotics group (4.7 days on average vs. 10.5 days, *p* = 0.03). The vaginal microbiota composition during pregnancy and after childbirth showed no significant differences between groups while the infants’ gut microbiome demonstrated a significantly higher abundance/prevalence of beneficial taxa in the probiotics group. The benefits conferred by probiotics were especially notable when considering birth by C-section. Probiotics promoted the vertical transmission of beneficial species and the induction of a highly interconnected microbiota, structured around key species. **Conclusions:** Probiotic supplementation during the third trimester of pregnancy and lactation is a valid strategy for conferring benefits to mothers and infants.

## 1. Introduction

Pregnancy involves physiological, hormonal, metabolic, and immune changes to accommodate the growing fetus [1,2]. Changes in the composition of the oral, gut, and vaginal microbiome also occur during this period [3,4,5]. Not only do these multiple changes make women more vulnerable to bacterial, viral, and fungal infections during pregnancy, but they also increase the severity of these infections [6,7]. The risk of urogenital infections such as vulvovaginal candidiasis (VVC), bacterial vaginosis (BV), and urinary tract infections (UTIs) is also increased during pregnancy [8,9,10,11]. Typical treatments such as antibiotics or antifungals can also affect the composition of the microbiomes, causing dysbiosis [12,13,14]. Dysbiosis in various body ecosystems during pregnancy has been associated with complications such as gingivitis, gestational diabetes, pre-eclampsia, and pre-term delivery [1,3].

Changes in the microbiome not only affect the mother’s metabolism but also play a role in the immune and metabolic health and development of the infant [3,4,5]. In fact, the infant’s gut microbiome is formed, in part, from microbes transmitted vertically from the mother’s various microbiomes, such as those of the intestine, skin, oral cavity, vagina, and breast milk [15,16].

The use of probiotics is an emerging strategy for the regulation of changes in the microbiome and the reduction in complications during pregnancy and post-partum [3,4]. Probiotics are defined as “live microorganisms that, when administrated in adequate amounts, confer a health benefit on the host” [17,18]. Probiotics can positively influence the composition of the intestinal microflora and exert various benefits, including improving immune function through their interaction with various immune cells [19]. In pregnant women, probiotic supplementation has been shown to improve immunological aspects as well as vaginal health [3,20,21,22]. Several studies have shown the positive effects of probiotics in preventing gestational diabetes [23,24] and reducing the risk of pre-eclampsia [25,26]. The use of probiotics could also provide beneficial microbial exposure to the newborn during delivery and breastfeeding [27], potentially extending the benefits of probiotics to the newborn’s health [28,29,30,31,32,33]. The current randomized, double-blind, placebo-controlled trial is one of the first and largest studies to investigate the effect of perinatal probiotics use on the mother’s immune system and protection against infection during pregnancy, as well as on the infant’s immune system and health status in the first year of life. The study also investigated the effects of probiotics consumption during pregnancy and breastfeeding on the establishment of the infant’s microbiome.

## 2. Methods

This clinical trial is reported according to the principles laid out in the Consolidated Standards of Reporting Trials (CONSORT) 2010 statement [34].

### 2.1. Study Approval and Registration

The study was conducted in accordance with the International Council for Harmonisation of Technical Requirements for Pharmaceuticals for Human Use guideline for Good Clinical Practice (ICH-GCP), and the ethical principles from the Declaration of Helsinki, and was approved by an independent Institutional Review Board, Veritas IRB (Saint-Laurent, QC, Canada) on 21 September 2017. This study was registered with ClinicalTrials.gov, identifier NCT03310853. All participants provided written informed consent prior to their participation in the study.

### 2.2. Participant Selection

Healthy pregnant women who were less than 28 weeks pregnant and aged between 18 and 39 inclusively were recruited at the Ovo Clinic (Montreal, QC, Canada). Inclusion criteria included singleton gestation, planning to breastfeed, and being willing to discontinue consumption of fermented foods (e.g., foods that contain probiotics such as Kefir, pickles, etc.) and probiotics (e.g., yogurts with live active cultures or probiotic supplements). Women could not participate in the study if they had a history of medical conditions such as HIV, Hepatitis (B or C), diabetes (gestational, type 1, or type 2), neurological or gastrointestinal diseases, and allergies to milk, soy, or yeast. Women with a personal history of (or diagnosed with) pre-eclampsia, maternal history of second trimester loss, and known fetal abnormality were also excluded. Symptoms of depression experienced in the past two years, smoking, alcohol consumption, drug use during pregnancy, or history of alcohol or substance use six months prior to screening were also exclusion criteria. A participant could not be included in the study if they had taken daily probiotics within 2 weeks of the visit or antibiotics within 1 month of the visit. However, they were eligible after a two-week washout period. After trial commencement but before enrollment of participants, the following exclusion criteria were added to the protocol to ensure participant safety and for their potential impact on trial outcomes: known blood/bleeding disorder, known liver or kidney disorders, and known unstable cardiovascular disease.

### 2.3. Study Design and Intervention

A randomized, double-blind, placebo-controlled, parallel-arm study was conducted to determine the effect of probiotics on the risk of infection in pregnant women and their newborns and on their microbiomes, and 180 participants were randomly assigned to the two arms in a 1:1 ratio. In the protocol, participants were supposed to be stratified based on the intended type of delivery (vaginal or C-section). This was not implemented due to the low number of planned C-sections. The randomization scheme used a random number generator available in JMP^®^ Pro 12.2 (SAS Institute, Inc., Cary, NC, USA). The random allocation sequence was generated by a statistician. Codes associated with the investigational products’ lot numbers were used for each group in the event that an adverse reaction required unblinding. Each participant who met inclusion criteria after screening by study personnel was given the study kit allocated in chronological order of her inclusion in the study; no number was omitted or skipped. The principal investigator and study staff were responsible for dispensing the product to participants according to the randomization schedule provided to site personnel. The sponsor representative involved in the study, the investigators, the participants, and any healthcare professional or site personnel involved in participant management or outcome assessment remained blind to allocation throughout the study. In addition, statisticians and biostatisticians who conducted the analysis were blinded to group allocation.

One of the randomized groups received a probiotic consisting of 5 × 10^9^ CFU of *Lacticaseibacillus rhamnosus* Rosell^®^-11 and *Bifidobacterium bifidum* HA-132 (Prenatis™), while the other group received a placebo. The placebo capsule was made of the same excipients as those used in the probiotic capsule and had a similar appearance. Both groups were instructed to take one capsule per day for approximately 18 weeks, i.e., 12 weeks prepartum and 4–6 weeks post-partum. The study consisted of 8 visits, with a prescreening, recruitment, and randomization visits, 5 interim visits, and a final end-of-study visit. In-person study visits were conducted at the Ovo Clinic, Montreal, Canada. The end-of-study visit was conducted over the phone. Assessments included blood tests, urine samples, capillary glycemia, vital signs, weight, saliva samples, vaginal swabs, stool samples for both mothers and infants, breast milk samples, and anthropometric measurements, as well as the completion of the Edinburgh Postnatal Depression Scale (EPDS), demographic questionnaires, mother and infant daily journals, one-year post-partum follow up questionnaires, and the collection of hospital data.

### 2.4. Outcomes

The primary outcome was the number of diagnosed infections in pregnant women. Secondary outcomes focused on both mothers’ and infants’ health. Secondary outcomes related to mothers’ health included levels of glucose, insulin, triglycerides, and ferritin measured in blood samples; inflammatory markers in blood, stool, and breast milk samples; levels of secretory immunoglobulin A (sIgA) in saliva, stool, and breast milk samples; vaginal microbiota and mycobiota composition; gut microbiota composition; breast milk microbiota composition; incidence of premature rupture of membranes (PROM); weight; stool frequency and consistency (Bristol Stool Form Scale, [35]); and post-partum depression (EPDS) [36]. Some secondary outcomes were removed from the original protocol to reduce participant burden: stress, anxiety, headache/migraine, and nausea self-reported daily as a diary entry; and collection of vaginal swabs at visit 3 and 4. Secondary outcomes related to infants’ health included the number of infections; levels of sIgA in stool samples; gut microbiota composition; stool frequency and pattern (Amsterdam Infant Stool Form Scale [37]); incidence of necrotizing enterocolitis (NEC); anthropometric measurements: crown–heel length and head circumference; weight; crying time and incidence of colic; sleep duration; skin diseases or conditions; and incidence of jaundice and hyperbilirubinemia. The duration of infections in mothers and infants, vertical transmission of microbiota in mother–infant dyads, and characterization of microbiota networks were exploratory outcomes defined after protocol registration.

### 2.5. Data Collection

Blood test, capillary glycemia, weight, saliva samples, vaginal swabs, stool samples for mothers and infants, breast milk samples, and anthropometrics measurements (infant) were performed during visits. Saliva and capillary blood glucose samples were collected only during prenatal visits. Participants also completed the EPDS questionnaire, demographic questionnaires, and maternal/infant daily diaries. On the last visit, all hospital data were collected. Lastly, a one-year post-partum follow-up was conducted over the phone.

### 2.6. Blood Inflammatory Markers

Blood samples were drawn through a venous catheter during visits 1 and 5 to test inflammatory cytokines. A custom 7-plex kit (Bio-Rad, Saint-Laurent, QC, Canada) was used for the multiplex immunoassay technique which contains—IL-1B, IL-6, IL-8, IFN-γ, and TNF-α (Custom Bio-Plex Pro Human Cytokine 7-plex, Bio-Rad, Hercules, CA, USA). The graphs were generated using GraphPad Prism version 9.5.1 (GraphPad software, Inc., San Diego, CA, USA).

### 2.7. sIgA Analysis

Salivary sIgA levels were measured from samples provided at visits 2, 3, 4, and 5. Stool sIgA levels were measured from samples provided at visits 2, 5, and 7. Breast milk sIgA levels were measured from the participants that provided breast milk samples at visit 7. The sIgA analysis were quantified using the Salivary Secretory IgA ELISA kits manufactured by Salimetrics (Carlsbad, CA, USA). The graphs were generated using GraphPad Prism version 9.5.1.

### 2.8. Microbiota Composition

Vaginal swabs and stool samples were collected at visits 2, 5, and 7. Breast milk samples were collected at visit 7. All samples were stored at −80 °C. The bacterial DNA extracted from the breast milk samples (using the DNeasy PowerSoil Pro Kit, QIAGEN, Hilden, Germany), the vaginal swabs (using the MasterPure Yeast DNA Purification Kit, Biosearch Technologies, Teddington, UK), and the stool samples (using the ZymoBiomics 96 MagBead DNA Kit, Zymo Research, Irvine, CA, USA) was used for microbiota profiling via 16S ribosomalRNA V3-V4 region amplicon sequencing following Illumina’s protocol. Briefly, the libraries were prepared using universal 16S primers (forward 5′-CCTACGGGNGGCWGCAG-3′ and reverse 5′-GACTACHVGGGTATCTAATCC-3′) for initial 16S amplification and Illumina Nextera XT V2 primers sets A, B, C, and D (Illumina, cat # FC-131-2001 FC-131-2002, FC-131-2003, and FC-131-2004) for the second PCR for multiplexing. The parallel sequencing was performed at 8 pM on a MiSeq platform using the MiSeq Reagent 600 cycles v3 Kit cartridge (Illumina, cat # MS-102-3003) and processed for 2 × 300 bp reads. The 16S amplicon sequencing results were exported in fastq files and imported into QIIME 2. The forward reads were inspected for quality and trimmed at 280 bp and reverse reads were trimmed at 260 bp (since the quality remained high throughout). For vaginal microbiota composition and its association with BV, the reads were quality filtered using QIIME™’s dedicated module and were used to generate (cluster the sequences) and count the amplicon sequence variants (ASVs) present in each sample [38]. Taxonomic profiles were generated for each sample by taxonomic attribution of the ASVs using the QIIME™ 2 feature-classifier machine-learning-based tool and the database, SILVA 138. The taxonomic profiles for Baseline, Probiotic, and Placebo were generated and compared globally on group averages and on individual taxa and strains. For the proportions of vaginotypes and association with BV and antibiotic use, and for breast milk and infants’ gut microbiome, the targeted sequences from microbiota were analyzed using a bioinformatic pipeline based on Dadaist2 software v1.2.5 [39]. Briefly, after demultiplexing the barcoded Illumina paired reads, single read sequences were paired for each sample into longer fragments and cleaned. After quality-filtering and sequencing error modeling, ASVs were obtained. A taxonomic assignment of these ASV was performed on RDP database release 18 to determine bacterial community profiles.

### 2.9. Sample Size Calculation

Sample size calculation was performed based on the likelihood of recurrence of specific types of infections such as VVC, BV, and self-reported colds/flu [40,41,42,43,44]. Hilton et al. [44] found that, in a cross-over design with a small number of subjects (*n* = 13) with ≥5 VVC events/year, the mean number of infections while on probiotics for six months was 0.38 ± 0.51 compared to 2.54 ± 1.16 while not on probiotics. Langkamp-Henken et al. [41] reported that the number of episodes of self-reported colds/flu during a six-week study of stressed college students (*n* = 150/treatment) varied by probiotic use. Mean number of colds/flus was 0.63 ± 0.07 on placebo; 0.61 ± 0.07 on *L. helveticus*; 0.43 ± 0.06 on *B. bifidum*; and 0.48 ± 0.06 on *B. longum* ssp. *infantis*. Based on the findings reported by Hilton et al. [44], mean number of infections was estimated to be 1 for the probiotics group and 2 for the placebo group. The variance estimates for the probiotics group was 1.5 and 3 for the placebo group. Other parameters used for this calculation were a difference in means of 1 event with pooled standard deviation of 2.12, two-sided test, desired power of 0.8, and type I error rate of α = 0.05. The resulting calculation gave a sample size per arm of 71. To allow for a 20% dropout rate, the initial size was 89 per arm or 178 in total, which was rounded up to 180 participants.

### 2.10. Statistical Analysis

Statistical analyses were conducted with the Statistical Analysis System (SAS, v9.4) and R version 4.3.3. The primary outcome (number of diagnosed infections in mothers) was analyzed with a negative binomial regression. Other count variables representing rare events compiled over the duration of the study were analyzed with this test. Single time-point measurements and differences between groups at baseline were compared using the Wilcoxon signed-rank test for continuous variables and Fisher’s exact test for presence/absence (percentages) variables. All analyses used a Type I error rate of α = 0.05.

Methods for vertical transmission analysis are augmented from the approach described by Mortensen et al. [45]. Univariate analysis was performed to identify which taxa are transmitted from one of the mother’s microbiomes to the child’s intestinal microbiome. For each taxon, the odds ratio (OR) and *p*-value for transmission from mothers’ microbiota to their child’s gut microbiota was calculated. *p*-values were then FDR-adjusted. To pursue an enrichment hypothesis, a Weighted Transfer Ratio (WTR) was calculated. WTR is used as an overall measure of transfer. It is based on the OR and *p*-value for each individual ASV and calculated as the weighted ratio of positive OR (OR > 1) and negative OR (OR < 1), with WTR > 1 indicating enrichment and WTR < 1 indicating depletion of positive OR, defined as follows:

$$WTR=\frac{\sum_{i\in I\left(OR>1\right)}-\log\left(OR_{i}\right)\log_{10}\left(pv_{i}\right)}{\sum_{i\in I\left(OR<1\right)}\log\left(OR_{i}\right)\log_{10}\left(pv_{i}\right)}$$

To estimate the significance of the WTR, 10,000 permutations consisting of randomly associating a mother and a child were carried out. To study the effect of probiotics on vertical transfer, the difference in WTRs calculated within each group was compared to the distribution of differences in WTRs randomly associating individuals to a group.

### 2.11. Exploratory Analysis of Bacterial Networks

Estimation of microbial networks was performed using SPIEC-EASI (Sparse Inverse Covariance Estimation for Ecological Association Inference, Kurtz et al. [46]). This computational method is designed to robustly infer microbial community networks from high-dimensional microbiome data. By leveraging sparse inverse covariance estimation and a bootstrapping stability analysis, SPIEC-EASI identifies and estimates relevant conditional dependencies between microbial species, facilitating the construction of networks that reveal underlying ecological interactions. Networks inferred using SPIEC-EASI should be interpreted as simplified models of the potential ecological interactions between microbial species within the community. Nodes represent individual microbial taxa, while edges indicate conditional dependencies between them. The presence of an edge implies that the relationship between two taxa is not solely driven by other taxa in the dataset, providing insights into direct associations within the microbiome. Conversely, the absence of an edge between two taxa indicates that any correlation between those two taxa is indeed not direct but completely driven by other components of the microbiota. While SPIEC-EASI is designed to minimize false positives through regularization and stability analysis, the presence of an edge does not confirm a causal relationship nor the type of association (i.e., competition or mutualism). Additionally, the sparsity induced by the method means that only the strongest associations are retained, potentially omitting weaker but biologically significant interactions.

## 3. Results

### 3.1. Participant Characteristics

**Figure 1:**
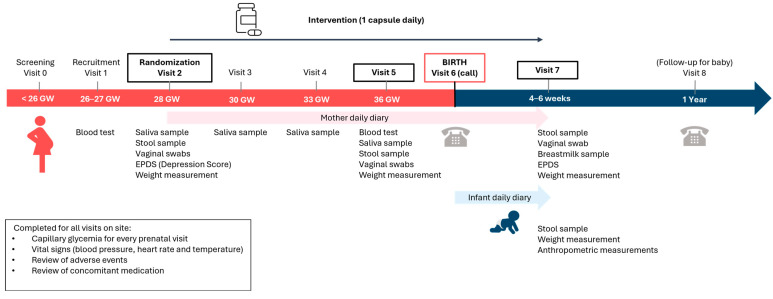
Study design. The study was a randomized, double-blind, placebo-controlled, parallel-arm trial spanning an 18-week period. The sample size for this study was 201 participants enrolled, with 180 participants randomized in a 1:1 ratio in each of the 2 arms. This study consisted of 8 visits, with prescreening, recruitment, and randomization visits, 5 interim visits, and a final end-of-study visit. To evaluate the primary and secondary outcomes, study assessments were conducted in 8 visits.

The study was initiated on 22 December 2017, and was completed on 21 July 2021. Over this period, a total of 180 women were enrolled in the study and were randomized into the probiotic (*n* = 90) or placebo group (*n* = 90). The study process is described in a flowchart, from the screening visit to the final study visit (Figure 1).

**Figure 2:**
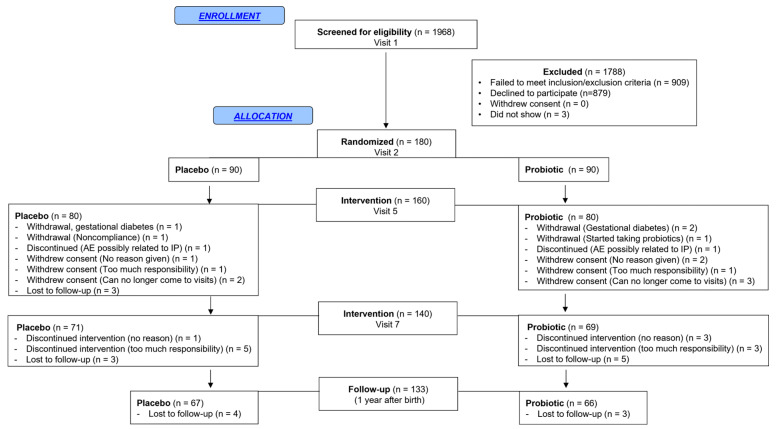
Flow diagram of study participants. Participants were recruited using various methods. A total of 1968 subjects were screened, and 180 were deemed eligible and randomized. Of the 180 participants randomized, 90 participants were allocated to the placebo group, and 90 participants were allocated to the study group to receive one capsule of probiotic supplement daily containing 5 × 10^9^ CFU of *Lacticaseibacillus rhamnosus* Rosell^®^-11 and *Bifidobacterium bifidum* HA-132 (Prenatis™). A total of 140 participants completed the 18-week study duration (71 in the placebo and 69 in the probiotics group), and 133 participants completed the follow-up one year after birth (67 in the placebo and 66 in the probiotics group). AE: adverse event; IP: investigational product.

The participants’ flow is presented in Figure 2. The compliance rate, determined based on the returned study product, was over 90% in both groups. A total of 123 unique participants experienced adverse events (AEs) (e.g., nasal congestion, headaches, constipation, diarrhea, etc.) with no significant difference in the mean number of AEs per participant in each group (probiotic: 1.41 ± 1.54; and placebo: 1.52 ± 1.38; *p* = 0.37). Participation was discontinued for three participants (one in the placebo group, and two in the probiotics group) due to gestational diabetes. Two participants (one in each group) decided to stop participating in the study due to AEs that they thought were related to the study products (abdominal bloating and occasional diarrhea). Eight participants (six in the placebo group and two in the probiotics group) experienced serious adverse events, but none were related to the trial.

Regarding infants, there was no pre-term birth in the placebo group and one in the probiotics group (Fisher’s exact test, *p* = 0.49), and no incidence of necrotizing enterocolitis in either group. The baseline characteristics of pregnant women and their infants at the time of birth are reported in Table 1 and show that the two groups are comparable.

Other characteristics of mothers and infants that were defined as secondary outcomes are presented as supplementary material (outcomes related to mothers’ health: levels of glucose, insulin, triglycerides, and ferritin—Supplementary Table S1; stool frequency and consistency—Supplementary Table S2; incidence of premature rupture of membranes (PROM)—Supplementary Table S3; change in weight over time—Supplementary Table S4; post-partum depression—Supplementary Table S5; outcomes related to infants’ health: crying time and colic—Supplementary Table S6; incidence of jaundice and hyperbilirubinemia—Supplementary Table S7; hours of sleep—Supplementary Table S8; stool frequency and pattern—Supplementary Table S9; skin diseases—Supplementary Table S10; and health problems—Supplementary Table S11).

### 3.2. Primary Outcome: Infections in Mothers

To evaluate the impact of probiotics on infections, participants in both groups were instructed to report any infections diagnosed by a physician or confirmed through laboratory tests during the third trimester. Reported infections included vaginal yeast infections, BV, sinusitis, bronchitis and conjunctivitis. Participants in the probiotics group tended to have a lower number of infections than participants in the placebo group, with a difference that approached statistical significance (*p* = 0.07) (Table 2). There was a lower mean number of BV per participant in the probiotics group (0.07 ± 0.29) compared to those in the placebo group (0.13 ± 0.37), but the difference was not significant (*p* = 0.19). There was no significant difference in the number of days with infections (*p* = 0.76).

**Figure 3:**
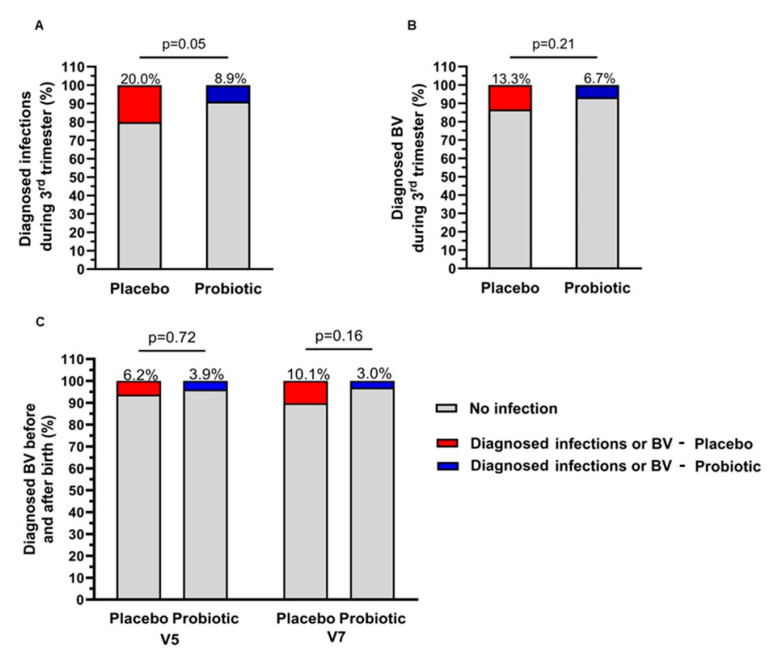
Proportion of participants with diagnosed infections. (**A**) Proportion of participants with diagnosed bacterial and fungal infections in the two groups during the 3rd trimester. Effect size (risk difference): −0.385; relative risk: −0.556. (**B**) Proportion of participants with bacterial vaginoses, diagnosed in the two groups during the 3rd trimester. Effect size (risk difference): −0.333; relative risk: −0.5. (**C**) Proportion of participants with diagnosed bacterial vaginosis in the two groups before (V5) and after (V7) birth. Fisher’s exact test was performed to determine the statistical differences between the placebo group (*n* = 90) and probiotics group (*n* = 90). V5 Effect size (risk difference): 0.0235; relative risk: −0.423. V7 Effect size (risk difference): 0.0716; relative risk: −0.723. BV: bacterial vaginosis; V: visit.

Looking at the number of women in each group diagnosed with one or more infections over the duration of the study, the number of participants was significantly lower in the probiotics group (*n* = 8, 8.9%) than the placebo group (*n* = 18, 20.0%) (*p* = 0.05) (Figure 3A). Among the various types of infections diagnosed, BV was the most common in the studied population, with 12 (13.3%) diagnoses in the placebo group and 6 (6.7%) in the probiotics group during the third trimester (Figure 3B).

### 3.3. Infections in Infants

Participants were asked to report daily, from the day of delivery until visit 7 (4 to 6 weeks after birth), whether an infection had been recently diagnosed (on the same day) in the infant, or if the diagnosed infection was ongoing and had persisted for more than 24 h. During this period, 11 infants were diagnosed with an infection, 7 in the probiotics group and 4 in the placebo group (*p* = 0.37). The diagnosed infections included oral candidiasis (thrush), UTI, conjunctivitis, omphalitis (umbilical stump infection), and skin infections (diaper rash related to *Candida* yeast infection). The mean number of days with an infection in the infants of both groups was analyzed. Infants whose mother received probiotic supplementation were sick for significantly shorter periods of time compared to those whose mother received the placebo (mean of 4.7 days vs. 10.5 days; *p* = 0.03) (Table 3).

### 3.4. Inflammatory Markers and Immunoglobulin Levels

Inflammatory markers in the blood and immunoglobulins in the saliva, stool, and breast milk were also measured. For both groups, proinflammatory (IL-1β, IL-6, IL-8, Interferon γ [IFN-γ], and Tumor Necrosis Factor α [TNF-α]) cytokines were measured in blood samples collected at visits 1 and 5 (Supplementary Figure S1A–E). The inflammatory cytokine levels were not significantly different between the two groups (Il-1β: V1, *p* = 0.59; V5, *p* = 0.67. IL-6: V1, *p* = 0.41; V5, *p* = 0.51. IL-8: V1, *p* = 0.7; V5, *p* = 0.51. TNF-α: V1, *p* = 0.71; V5, *p* = 0.61. INF-γ: V1, *p* = 0.22; V5, *p* = 0.68). An analysis of saliva samples collected at visits 2, 3, 4, and 5 revealed no significant differences in secretory Immunoglobulin A (sIgA) levels between women taking probiotics and those not taking probiotics (Supplementary Figure S1F) (at V2, *p* = 0.98; V3, *p* = 0.76; V4, *p* = 0.14; and V5, *p* = 0.72). The stool immunoglobulin levels were higher in the probiotics group, but the differences were not statistically significant (*p* = 0.89 at V2, 0.87 at V5, and 0.63 at V7) (Supplementary Figure S1G). Breast milk samples were collected after delivery, at visit 7, and the sIgA levels were measured (Supplementary Figure S1H). Probiotics had no significant effect on the sIgA levels in breast milk (*p* = 0.22). Regarding the levels of sIgA in the infants’ stool samples, there was no significant difference between groups (*p* = 0.8) (Supplementary Figure S2).

### 3.5. Vaginal Microbiome and Its Association with Infections

**Figure 4:**
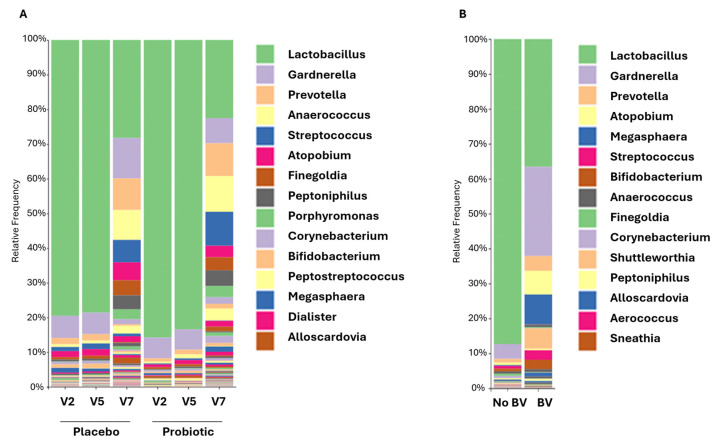
Vaginal microbiome and association with bacterial vaginosis. (**A**) Vaginal microbiome composition at genus level of the participants in the placebo and the probiotic groups at V2, V5, and V7. (**B**) Comparison of the vaginal microbiome of participants diagnosed with bacterial vaginosis during the 3rd trimester. The bar plots represent the relative abundance at genus level of vaginal microbiome compared at V2 between all participants having BV during pregnancy (BV) and participants not infected (No BV). BV: bacterial vaginosis; V: visit.

During pregnancy, the vaginal microbiome was characterized by the dominance of *Lactobacilli* (>70% relative abundance) in both the placebo and probiotics groups (Figure 4A). A minimal variation in the relative abundance of taxa was observed between visits 2 and 5.

Participants’ vaginal microbiome was categorized according to community state types (CSTs), also referred to as vaginotypes. CSTs represent five common configurations of vaginal microbiota taxonomic composition in women of reproductive age [47,48]. Four of those are dominated by a single species of *Lactobacillus:* CST I-*L. crispatus*, CST II-*L. gasseri*, CST III-*L. iners*, and CST V-*L. jensenii.* The last, CST IV, is characterized by the low abundance of *Lactobacillus* species and the presence of various facultative and obligate anaerobes (*Gardnerella*, *Atopobium/Fannyhessea*, *Prevotella*, *Sneathia*, *Peptoniphilus*, *Finegoldia*, and *Megasphaera*). While CSTs dominated by *Lactobacillus* species are associated with vaginal homeostasis, lower risks of urogenital infections, and better reproductive health, *L. iners* is regarded as the one that provides the least effective level of protection and is sometimes considered neutral in terms of its association with health outcomes [48]. CST IV could be seen as a state of disturbance of the vaginal flora, but it is important to note that many women with this vaginotype do not present any adverse vaginal symptoms. While the vaginal microbiome of women with CST IV can be considered as a dysbiotic ecosystem, this may not automatically lead them to experience abnormal vaginal discharge, itching, or burning sensations, which are associated with BV. Nevertheless, CST IV has been associated with greater risks of BV, sexually transmitted infections, and spontaneous preterm birth [48].

**Figure 5:**
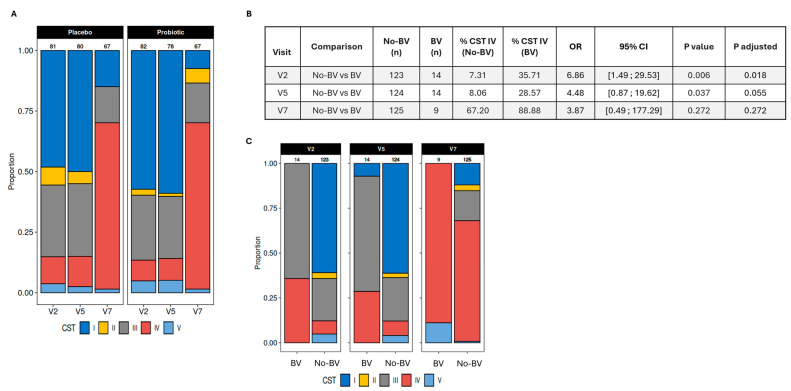
Proportions of vaginotypes and association with bacterial vaginosis. (**A**) Proportions of vaginotypes in the two groups at V2, V5, and V7. (**B**) The association of CST IV and bacterial vaginosis at V2, V5, and V7. Reported *p*-values correspond to Fisher’s exact tests and *p*-values adjusted for simultaneous testing using a Benjamini–Hochberg procedure. (**C**) Distribution of vaginotypes in participants with and without infections at V2, V5. and V7. BV: bacterial vaginosis; Inf: infections; No-inf: no infections; V: visit; CST: community state types; CST I: *Lactobacillus crispatus*; CST-II: *Lactobacillus gasseri*; CST-III: *Lactobacillus iners*; CST-IV: non-lactobacillus-abundant; and CST-V: *Lactobacillus jensenii*.

In the present study, CST distributions at visits 2 and 5 showed a higher relative abundance of CST I-*L. crispatus* (protective) and CST III-*L. iners* (neutral), and a lower relative abundance of CST IV (greater risks of infections), CST II-*L. gasseri* (protective), and CST V-*L. jensenii* (protective) in both groups (Figure 5A). While group differences were not statistically significant, the probiotics group had lower proportions of CST IV, which has been associated with a greater risk of infection, and CST III-*L. iners*, which provides the least effective protection of all four *Lactobacillus*-characterized CSTs (Figure 5A) [48]. Looking more specifically at participants’ vaginal microbiome according to the incidence of BV, a markedly lower relative abundance of *Lactobacilli* was observed in participants who reported a BV diagnosis at visits 2 or 5 (<40% relative abundance) compared to participants who did not (>80% relative abundance) (Figure 4B). The reduction in *Lactobacilli* was in favor of pathogenic or opportunistic pathogenic taxa such as *Gardnerella* and *Atopobium*, which are characteristic of CST IV. The proportion of participants who reported a BV at visit 5 was lower in the probiotics group (3.9%) than in the placebo group (6.2%), but this difference was not statistically significant (Figure 3C). The prevalence of CST IV in the study population was significantly associated with the incidence of BV at visit 2 and can be considered as a statistical trend at visit 5 (Figure 5B,C).

**Figure 6:**
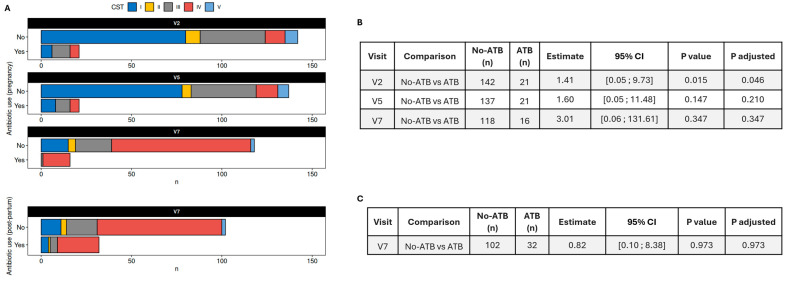
Proportions of vaginotypes and association with antibiotic use. (**A**) Proportions of vaginotypes in the participants taking antibiotics or not at V2, V5, and V7. (**B**) The association of vaginotypes and antibiotic use during pregnancy. (**C**) The association of vaginotypes and antibiotic use post-partum. Reported *p*-values correspond to Fisher’s exact tests and *p*-values adjusted for simultaneous testing using a Benjamini–Hochberg procedure. Effect size and CI are estimated with null cells removed from contingency tables. V: visit; CST: community state types; CST I: *Lactobacillus crispatus*; CST-II: *Lactobacillus gasseri*; CST-III: *Lactobacillus iners*; CST-IV: non-lactobacillus-abundant; CST-V: *Lactobacillus jensenii*; and ATB: antibiotic.

Participants were asked to report their use of antibiotics during the third trimester of pregnancy at visits 2, 5, and 7. The measurement taken at visit 7 accounted for their use of antibiotics between visit 5 and childbirth. Regarding the distribution of CST in relation to the use of antibiotics, greater proportions of CST IV (at visits 2, 5, and 7) and CST III-*L. iners* (at visits 2 and 5) were observed in participants who reported using antibiotics during pregnancy compared to those who did not (Figure 6A). CST III and IV were associated with an increased risk of infection that required the use of antibiotics at visit 2 (adjusted *p*-value = 0.046; effect size estimate = 1.41, 95% CI: 0.05–9.73) (Figure 6B).

After childbirth (visit 7), the vaginal microbiome profile showed a loss in the dominance of *Lactobacilli* (<30% relative abundance) associated with an increase in taxa that characterize CST IV, including *Gardnerella*, *Atopobium,* and *Anaerococcus* (Figure 4A). Changes in the relative abundance of taxa were reflected in the distribution of vaginotypes. At visit 7, CST IV was the most prevalent in both groups. While there was a higher proportion of CST II-*L. gasseri* in the placebo group at visits 2 and 5, this vaginotype was no longer present in this group after childbirth. However, it was still identified in the probiotics group (Figure 5A). There was no difference between the two groups at any of the visits, but the distribution of CSTs was significantly different before (visit 2–5) and after childbirth (visit 7) in both groups (all *p* values < 0.001). To assess whether probiotics had a beneficial effect on the protection against infections after childbirth, participants were asked at visit 7 to report any diagnoses of BV occurring since delivery. The diagnoses were confirmed by analyzing bacterial cultures from vaginal swabs. The results revealed a trend towards a lower prevalence of diagnosed BV in the probiotics group (3.0%) compared to the placebo group (10.1%) (*p* = 0.16) (Figure 3C). There was no association between vaginotypes observed at visit 7 and the use of antibiotics after childbirth (*p* = 0.97) (Figure 6C).

Lastly, the absolute quantification of fungal DNA in vaginal swabs was performed, but most samples showed levels lower than the detection threshold. Therefore, planned sequencing analysis to characterize vaginal mycobiota was not performed.

### 3.6. Mother and Infant Gut Microbiota

The recovery of probiotic strains in mothers’ and infants’ stool samples is reported in Supplementary Figure S3. Both strains were detected in more samples from mothers in the probiotics group and their infants than mothers in the placebo group and their infants. Mothers’ gut microbiota composition showed no particularities between groups both before (visit 2 and 5) and after birth (visit 7) (Supplementary Figure S4). Regarding breast milk, the microbiota composition showed a higher abundance of *Streptococcus* and *Bacillus*, and a lower abundance of *Staphylococcus* and *Pseudomonas* in the probiotics group compared to the placebo group (Supplementary Figure S5).

**Figure 7:**
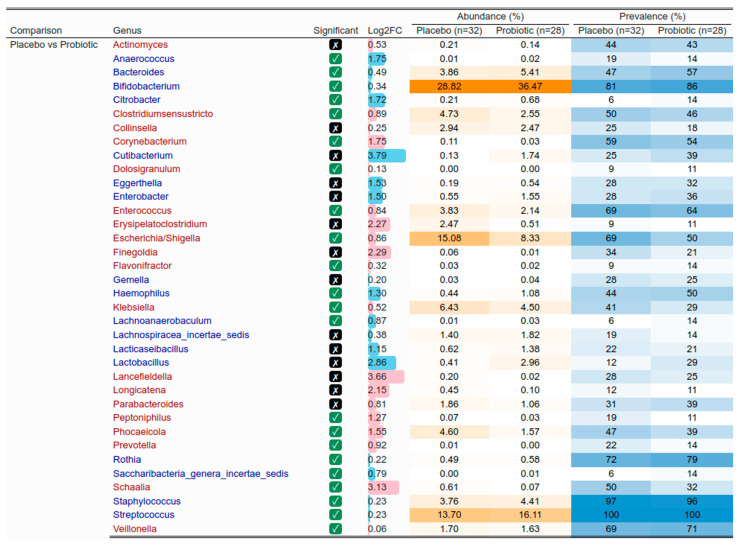
Gut microbiome of exclusively breastfed infants. Comparison of the abundance and prevalence of the most abundant taxa in exclusively breastfed infants in their first 4 to 6 weeks of life. Differential abundance analysis using ANCOM-BC method was performed to determine the statistical differences between the placebo group (*n* = 32) and probiotics group (*n* = 28). The taxa presented in blue or red are increased or decreased in the probiotics group, respectively. The intensity of orange and blue colors in the figure are proportional to the relative abundance and prevalence, respectively. Log2FC: log2 of the mean abundance difference between compared groups. The absolute value of Log2FC was clipped to a maximum of 5 to improve readability.

The relative abundance of taxa in the gut microbiota of exclusively breastfed infants showed significant differences between infants whose mothers took probiotic supplementation and infants whose mothers took the placebo (hereafter, infants in the probiotic and placebo group) (Figure 7). Some of those differences, but not all, were driven by prevalence, that is, the presence or absence of taxa. Notably, infants in the probiotics group had higher abundances of *Bifidobacteria*, *Streptococcus*, *Bacteroides*, and *Staphylococcus*, and lower abundances of *Escherichia/Shigella*, *Klebsiella*, *Enterococcus*, *Corynebacterium*, *Veillonella,* and *Prevotella*. Together, differences in the abundance of *Bifidobacteria* (considered beneficial) and *Escherichia/Shigella* and *Klebsiella* (opportunistic pathogens) indicated a more favorable profile in the probiotics group.

**Figure 8:**
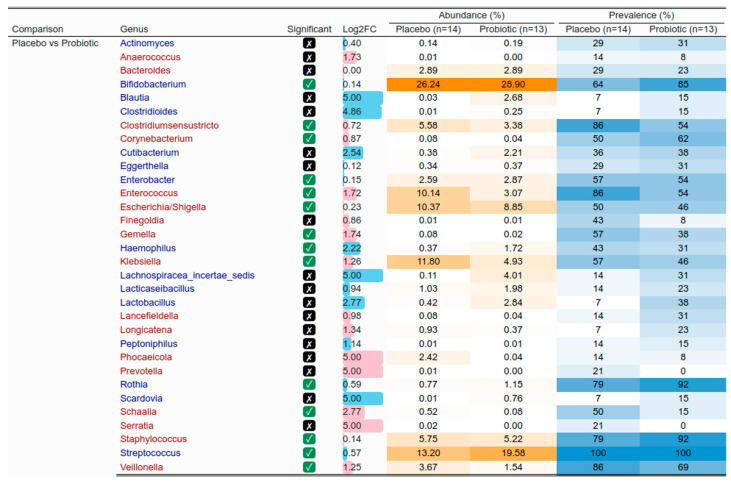
Gut microbiome of infants born by C-section. Comparison of the abundance and prevalence of the most abundant taxa in infants born by C-section in their first 4 to 6 weeks of life. Differential abundance analysis using ANCOM-BC method was performed to determine the statistical differences between the placebo group (*n* = 14) and probiotics group (*n* = 13). The taxa presented in blue or red are increased or decreased in the probiotics group, respectively. The intensity of orange and blue colors in the figure are proportional to the relative abundance and prevalence, respectively. Log2FC: log2 of the mean abundance difference between compared groups. The absolute value of Log2FC was clipped to a maximum of 5 to improve readability.

While group differences in the gut microbiota composition of infants born by C-section involved similar taxa, the variations were more pronounced, especially regarding prevalence (Figure 8). For instance, the significant difference in abundance of *Bifidobacteria* was reflected in a prevalence of 64% in the placebo group compared to 85% in the probiotics group. In the larger subset of exclusively breastfed infants, the abundance of *Bifidobacteria* was also significantly different between groups, but the prevalence was 81% in the placebo group compared to 86% in the probiotics group. The differences in the abundance of *Escherichia/Shigella*, *Klebsiella*, *Enterococcus,* and *Veillonella* are additional indicators of a more favorable profile in the probiotics group. Overall, the benefits of probiotics were more pronounced in infants born by C-section.

### 3.7. Vertical Transmission

**Figure 9:**
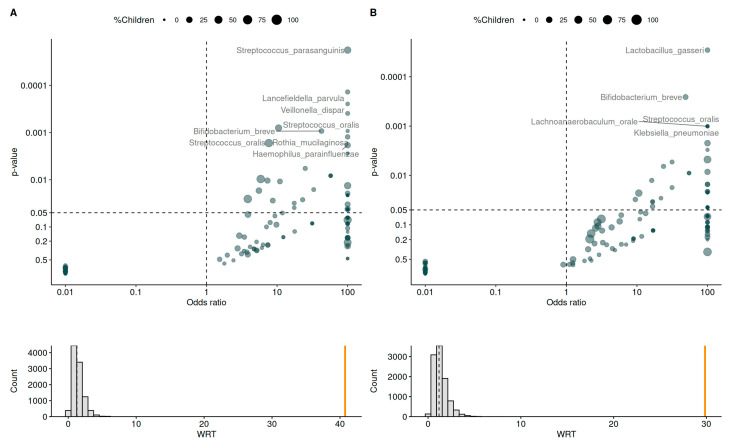
Vertical transfer through breastmilk. Effect of probiotics (**B**) on vertical transfer through breastmilk in comparison to placebo (**A**). Labeled taxa are the ones that are significantly transferred after statistical adjustment. Weighted Transfer Ratio (WTR) was calculated (based on the OR and *p*-value for each individual ASV and calculated as the weighted ratio of positive OR (OR > 1) and negative OR (OR < 1)). To estimate the significance of the WTR, 10,000 permutations consisting of randomly associating mother/child were carried out. Lower panels show the permutation distribution of the WTR statistic, with the observed value as an orange stem.

The analysis of the vertical transmission of the microbiota between the mother’s milk and the infant’s gut revealed the transmission of several taxa and different profiles across groups (Figure 9). After statistical adjustment, vertical transmission was significant for eight species in the placebo group (*Streptococcus parasanguinis*, *Lancefieldella parvula*, *Veillonella dispar*, *Streptococcus oralis*, *Bifidobacterium breve*, *Rothia mucilaginosa*, *Streptococcus oralis,* and *Haemophilus parainfluenzae*) and five species in the probiotics group (*Lactobacillus gasseri*, *Bifidobacterium breve*, *Streptococcus oralis*, *Lachnoanaerobaculum orale,* and *Klebsiella pneumoniae*). Among them, only *Bifidobacterium breve* (observed in the probiotic and placebo groups) and *Lactobacillus gasseri* (observed in the probiotics group only) are considered beneficial, with several probiotic strains from these species associated with various health benefits [49,50]. Other species are commensal bacteria from other ecosystems (skin, oral cavity, and upper respiratory tract) and may be considered opportunistic pathogens.

**Figure 10:**
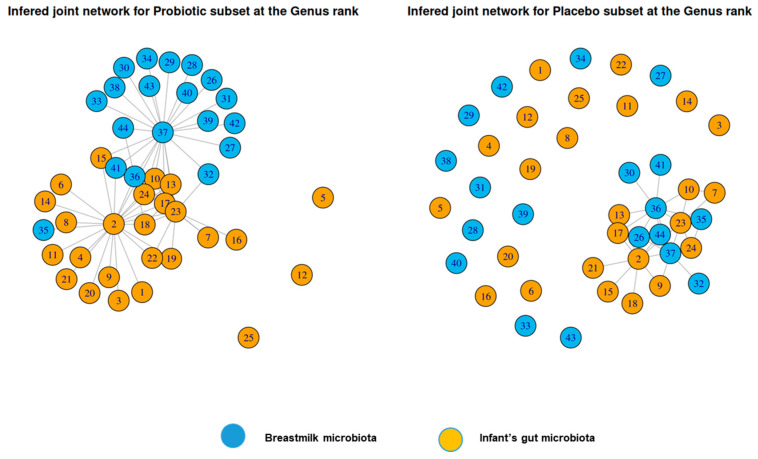
Bacterial networks of the breastmilk and infants’ gut microbiota. Inferred joint network at genus level of the breastmilk microbiota and infant’s gut microbiota in the two groups at V7. Compositional networks were inferred using sparse graphical models. V: visit. Infant’s gut microbiota: (1) *Schaalia*, (2) *Bifidobacterium*, (3) *Rothia*, (4) *Corynebacterium*, (5) *Cutibacterium*, (6) *Lancefieldella*, (7) *Collinsella*, (8) *Eggerthella*, (9) *Bacteroides*, (10) *Phocaeicola*, (11) *Parabacteroides*, (12) *Gemella*, (13) *Staphylococcus*, (14) *Dolosigranulum*, (15) *Enterococcus*, (16) *Lactiseibacillus*, (17) *Streptococcus*, (18) *Clostridiumsensustricto*, (19) *Lachnospiracea*, (20) *Finegoldia*, (21) *Veillonella*, (22) *Enterobacter*, (23) *Escherichia/Shigella*, (24) *Klebsiella*, (25) *Haemophilus*. Breastmilk microbiota: (26) *Bifidobacterium*, (27) *Intrasporangium*, (28) *Kocuria*, (29) *Micrococcus*, (30) *Rothia*, (31) *Corynebacterium*, (32) *Cutibacterium*, (33) *Prevotella*, (34) *Chryseobacterium*, (35) *Gemella*, (36) *Staphylococcus*, (37) *Streptococcus*, (38) *Anaerococcus*, (39) *Finegoldia*, (40) *Peptoniphilus*, (41) *Veillonella*, (42) *Brevundimonas*, (43) *Haemophilus*, and (44) *Acinetobacter*.

To further understand the influence of the mother’s microbiome on the induction and development of the microbiome in infants, network analyses of infants’ gut microbiota and mothers’ milk microbiota were conducted. When analyzing the microbiota composition at the genus level by considering co-occurrences instead of bacteria individually, bacterial networks of infants’ gut microbiota and mothers’ milk microbiota showed a stronger density of connections in the probiotics group than in the placebo group (Figure 10). Furthermore, in the probiotics group, connections were organized around key bacteria in each microbiota: *Bifidobacterium* in infants’ gut microbiota and *Streptococcus* in mothers’ milk microbiota. Only three bacterial genera were not included in the network, and these are mainly found in other ecosystems: the oral cavity (*Gemella*, *Haemophilus*) and the skin (*Cutibacterium*). In the placebo group, a rudimentary network with a low density of connections was observed, and twenty-five genera were left out of the network. Taken together, these results suggest that probiotics favor the induction of a structured microbiota, rather than the transmission of isolated bacteria. A toy example that supports the interpretation of network analysis is included in the supplementary material (Supplementary Figure S6).

## 4. Discussion

Pregnant women can be more susceptible to certain viral, bacterial, and fungal infections, and pregnancy can make these infections worse [7,8,51,52]. The main objective of this study was to assess whether probiotic supplementation during pregnancy could confer protective benefits against infections for both pregnant women and their infants, thereby potentially reducing the associated risks of these infections. Another objective of the study was to assess the potential beneficial effects of probiotic consumption during pregnancy and breastfeeding on the establishment of the infant’s microbiome.

The results indicated that probiotics supplementation during pregnancy reduced the incidence of both bacterial and fungal infections, and there was a significantly lower number of women diagnosed with infections during the study in the probiotics group. While there was a trend for the reduction in the number of infections in the probiotics group, the significant reduction in the proportion of women presenting infections over the course of the study supports the idea that probiotics were broadly effective and that their benefits were not only experienced by a subset of intervention responders. This is an interesting finding from a clinical perspective, considering the study’s main objective to test a supplement that confers protection against infections during the last trimester of pregnancy. Women who received probiotics tended to be less susceptible to bacterial vaginosis (BV), the most commonly observed infection, compared to those in the placebo group. Although it was not significant, this trend persisted post-partum, with a lower prevalence of diagnosed BV in the probiotics group relative to the placebo group. These findings suggest a potential benefit of probiotics in reducing the incidence of BV not only during pregnancy but also after childbirth. The literature provides substantial evidence supporting the beneficial effects of probiotics in protecting against and preventing different types of infections [43,53,54,55]. In pregnant women, studies have shown that probiotics reduce the colonization of certain bacteria, such as Group B *Streptococci*, thereby reducing the risk of infection associated with these bacteria [56,57]. Ang et al. have shown that probiotics had a beneficial effect against vaginal candidiasis in pregnant women through the modulation of the vaginal microbiota [58].

The presence of a vaginal microbiome that is strongly dominated by *Lactobacilli* during pregnancy is coherent with the observations of other studies conducted in pregnant women [59,60,61]. This dominance would be established during the first trimester and continue throughout pregnancy [59,60,61]. While the mechanisms behind *Lactobacilli* dominance during pregnancy are not fully understood, some researchers have suggested a link with elevated estrogen levels [59]. The marked changes in composition and diversity observed after childbirth align with the findings reported in the literature [62,63]. The steep decrease in the relative abundance of *Lactobacilli* and the increase in diverse anaerobes that characterize CST IV such as *Peptoniphilus*, *Prevotella*, *Atopobium,* and *Megasphaera,* are typical of changes in the vaginal microbiome observed post-partum [62,63]. As reported by others [62], the most common vaginotype observed after childbirth was CST IV. Interestingly, while CST II was observed in slightly larger, although not significantly different, proportions in the placebo group than the probiotics group during pregnancy, this vaginotype was no longer present in the placebo group but was still observed in the probiotics group after childbirth. CST II is characterized by the dominance of *Lactobacillus gasseri*, a species with strains that have recently been identified as potential probiotics for women [50,64]. Bae et al. [50] have suggested probiotic indications for *L. gasseri* LM1065 due to its capacity to inhibit candidiasis by suppressing the tricarboxylic acid cycle in *Candida albicans* and blocking its transition to hyphae. Zhang et al. [64] screened vaginal secretion and identified a *L. gasseri* strain (*L. gasseri* VHProbi E09) that was capable of inhibiting *Gardnerella vaginalis* and *Candida albicans,* which are associated with BV. Under co-culture conditions, *L. gasseri* VHProbi E09 could inhibit the growth of *G. vaginalis* and *C. albicans,* and significantly inhibit the adhesion of these pathogens to vaginal epithelial cells. It further demonstrated the ability to inhibit the enteropathogenic bacteria *Escherichia coli* and *Salmonella enteritidis*. In summary, *L. gasseri*, which is naturally present in human breast milk [50], would confer several health benefits on the host. In the present study, we have found that the significant vertical transmission of *L. gasseri* was only observed in infants of the probiotics group. These results will be further discussed below.

One of the mechanisms by which probiotics may protect against infections is their ability to modulate the immune system by communicating and interacting with various types of immune cells [65,66], and by participating in bile acid metabolism [67]. The immunomodulatory effects of probiotics would mainly be due to their ability to stimulate the production of cytokines such as interleukins, transforming growth factor (TGF), tumor necrosis factors (TNFs), and interferons, as well as chemokines from immune cells [65]. Our study found no differences in the levels of blood inflammatory markers (such as IL-1β, IL-6, IL-8, TNF-α, and IFN-γ) and sIgA in saliva, stool, and breast milk between women who received probiotics and those in the placebo group. The meta-analysis conducted by Maia et al. demonstrates that, although some studies have shown the modulation of blood inflammatory marker levels by probiotics, the results of other studies align with our observations regarding the absence of an effect of probiotics on these blood proinflammatory cytokines [68]. The modulation of the immune system by probiotics also involves their interaction with B cells, which can subsequently induce an increase in sIgA production [19,69]. Nevertheless, our results are consistent with part of the literature indicating that there is no significant effect of probiotics on salivary sIgA production [70]. In a study conducted by Prescott et al., the intake of probiotics by pregnant women for 2 to 5 weeks before delivery, and for a duration of 6 months, led to an increase in the level of sIgA in breast milk [71]. In contrast, consistent with our findings, other studies have not reported significant differences in sIgA levels in breast milk between individuals who took probiotics and those who did not [72,73]. The differences in results may be due to the various probiotic strains utilized, and the categories of participants, as well as the doses and duration of supplementation.

Probiotic intake during pregnancy is known to have effects on infants, with evidence showing that maternal probiotic consumption plays a role in the colonization and modulation of the infant’s microbiome [27,32,74,75]. To evaluate the potential beneficial effects of probiotic intake by women during pregnancy and breastfeeding on infants, we assessed infections in the infants and analyzed their microbiomes. We did not observe a difference in the number of infections between the two groups of infants, but the number of days with infections was significantly lower in the probiotics group, with an average of 4.7 days of infection compared to 10.5 days on average in the placebo group. This supports an extension of the benefits observed in mothers to their infant.

The analysis of the gut microbiota composition of exclusively breastfed infants revealed interesting differences between the groups. Specifically, infants in the probiotics group exhibited higher abundances of *Bifidobacteria*, *Streptococcus*, *Bacteroides*, and *Staphylococcus*, and lower abundances of *Escherichia/Shigella*, *Klebsiella*, *Enterococcus*, *Corynebacterium*, *Veillonella,* and *Prevotella* compared to infants in the placebo group. The higher abundance of beneficial bacteria (e.g., *Bifidobacteria*), lower abundance of pathogens or opportunistic pathogens (e.g., *Escherichia/Shigella*), and patterns involving antagonistic bacteria (e.g., higher abundance of *Bacteroides* with lower abundance of *Prevotella* [76]) suggest a more favorable profile in the probiotics group. *Bifidobacteria* are among the first colonizers of the infant gut and dominate the composition of the early gut microbiota, especially in exclusively breastfed infants [77,78,79]. Various perinatal events, notably birth by C-section, can affect the gut colonization by *Bifidobacteria* [77,78,79,80]. In infants born by C-section, the lack of exposure to bacteria present in the birth canal alters the development of the gut microbiome [81,82]. In the present study, we observed more marked differences in the gut microbiota of infants in the probiotics group compared to those in the placebo group among infants born by C-section. Similar to our findings in exclusively breastfed infants, we noted a higher abundance of *Bifidobacteria* in the probiotics group. However, the difference in prevalence of *Bifidobacteria* was much greater when focusing on the subset of infants born by C-section (probiotic: 85%; placebo: 64%; 21% difference) than when analyzing the larger subset of data from infants who were exclusively breastfed (probiotic: 86%; placebo: 81%; 5% difference). Promoting gut colonization by *Bifidobacteria* in infants born by C-section is an important clinical indication with several implications for health outcomes in early infancy and later in life [78,79,80,81]. This is thought to act preventively on outcomes associated with birth by C-section, including an increased risk of obesity, metabolic diseases, and altered immunological development [81]. Vaginal seeding has been used to restore the development of the gut microbiome in infants born by C-section [81,83]. This procedure consists of incubating a sterile gauze in the mother’s vagina for one hour before the C-section, and swabbing the newborn’s mouth, face, and rest of the body with the gauze within the first minutes after birth [83]. Vaginal seeding can help initiate the development of a similar microbiome to the one observed in infants born vaginally in infants born by C-section [81,83]. However, this procedure presents inherent risks of pathogen transmission, and, therefore, providing probiotic supplements to mothers during lactation has been suggested as a safer alternative [81]. In the present study, probiotics taken by the mother in the third trimester of pregnancy and during lactation promoted gut colonization by *Bifidobacteria* in infants born by C-section, as supported by the significant differences between groups in terms of the relative abundance and prevalence of this taxa. Our results are in line with a review that concluded that infants born by C-section who receive probiotics experience a greater benefit from the intervention compared to those who are born vaginally, since probiotics help compensate for the lack of exposure to the mother’s microbiota during the passage through the birth canal [79]. This finding has important clinical implications in view of the increase in the incidence of birth by C-section observed worldwide [84].

Vertical transmission involved more species in the placebo group than in the probiotics group, but only one of the species with significant transmission in the placebo group (*Bifidobacterium breve*) can be considered beneficial [49], while others are mostly opportunistic pathogens. In the probiotics group, the significant transmission of both *Bifidobacterium breve* and *Lactobacillus gasseri* was observed. As discussed previously, *L. gasseri* is found in breast milk and has several probiotic properties that can be beneficial to both mothers and infants [50,64]. Beyond the transmission of isolated bacteria species, this study highlighted the transmission of a structured and more densely connected network of bacteria in the probiotics group than in the placebo group. Importantly, in the probiotics group, networks were structured around key bacteria in each microbiome: *Bifidobacterium* in infants’ gut microbiota and *Streptococcus* in mothers’ milk microbiota. A recent study focusing on the development of the gut microbiota in infants who were born prematurely found that the microbiome of infants who received a probiotic transitioned more rapidly into a state resembling that of infants born at term, which was characterized by higher stability and species interconnectivity [85]. High interconnectivity is a desirable feature of the infant gut microbiome, since human microbiomes are networks showing a strong organization with a few “hub” taxa driving the composition of each ecosystem [86]. While it is strongly dominated by *Bifidobacteria* and, hence, much less diverse than the adult gut microbiome, the infant gut microbiome may perform its functional requirements by relying on a highly interconnected network of species with strong metabolic specialization [78]. Based on our results, it appears that the administration of probiotics supplements to mothers in the last trimester of pregnancy and during lactation would promote the induction of a more interconnected network of gut bacteria in infants.

While this study has strengths, including the analysis of the mothers’ milk and infant gut microbiota, as well as the analysis of vertical transmission in mother–infant dyads, it also has some limitations. The overall number of infections reported by mothers was small. Healthy participants were recruited for this study, but it may have been relevant to recruit participants with a recent history of infections or recurrent infections to better appraise the effect of the probiotic. For safety reasons, participants with diabetes (gestational, type 1, and type 2) were excluded from the present study. However, other studies have shown that probiotics can prevent or help control gestational diabetes [23,24]. Now that there is evidence for the safety and efficacy of the Prenatis™ probiotic supplement, future studies could recruit participants with gestational diabetes specifically or allow participants with this condition to take part in the study. This would be a relevant contribution to knowledge, considering that women with gestational diabetes are more susceptible to some infections [87] and may, therefore, benefit from taking a probiotic supplement. Other complementary analyses could have provided relevant information to better understand the modulation of the microbiota composition by probiotics, such as measures of pH from vaginal swabs and the human milk oligosaccharide (HMO) composition of mothers’ milk. In addition to collecting a stool sample one month after birth, the collection of samples earlier and later in the infants’ life would have allowed us to track changes in the composition of infants’ gut microbiota more precisely and to assess if group differences were maintained over time. Similarly, the collection of vaginal swabs during the first months or year after birth would have allowed us to track the restoration of the vaginal microbiota over time.

## 5. Conclusions

In conclusion, the consumption of the Prenatis™ probiotic supplement during the third trimester of pregnancy contributes to reducing the rate of infections in expecting mothers. The benefits extend to their infants and include a shorter duration of infections, enhanced gut colonization by beneficial bacteria (especially *Bifidobacteria*), and the induction of a highly interconnected microbiota that is structured around key species, which may confer health benefits in early infancy and throughout life. Since they lack exposure to the mother’s vaginal microbiota and may require additional support to initiate healthy microbiome establishment, infants born by C-section may benefit from probiotic intake in mothers during pregnancy and lactation to a larger extent than infants born vaginally.

## Figures and Tables

**Table 1:** Baseline characteristics of women receiving probiotics or placebo and their infant.

	Placebo	Probiotics	*p*-Value
Women (*n*)	90	90	
Age in years (Mean ± SD)	30.4 ± 4.0	31.2 ± 4.0	0.26
Height in cm (Mean ± SD)	163.9 ± 6.0	163.5 ± 7.0	0.63
Weight in kg (Mean ± SD)	78.1 ± 15.0	78.3 ± 13.0	0.62
Infants (*n*)	71	69	
Gestational age at birth in weeks (Mean ± SD)	39.8 ± 1.3	39.7 ± 1.2	0.78
Weight at birth in kg (Mean ± SD)	3.4 ± 0.4	3.4 ± 0.5	0.93
Length at birth in cm (Mean ± SD) ^a^	50.3 ± 2.4	50.2 ± 2.4	0.88
Head circumference at birth in cm (Mean ± SD) ^b^	34.5 ± 1.4	34.7 ± 1.5	0.92
Born by C-section	17	15	0.84

^a^: placebo *n* = 71, probiotic *n* = 68; ^b^: placebo *n* = 67, probiotic *n* = 63. Born by C-section: Fisher’s exact test. All other comparisons: Wilcoxon signed-rank test.

**Table 2:** Infections in the third trimester of pregnancy.

	Placebo	Probiotics	*p*-Value	Effect Size	95% Confidence Interval	
	*n* = 90	*n* = 90			Lower	Upper
All Diagnosed Infections (Mean ± SD)	0.20 ± 0.45	0.09 ± 0.32	0.07	0.278	−0.052	1.673
Diagnosed BV (Mean ± SD)	0.13 ± 0.37	0.07 ± 0.29	0.19	0.226	−0.344	1.731
Days with Infections (Mean ± SD)	14.83 ± 7.93	13.75 ± 9.07	0.76	0.14	−0.428	0.58

Negative binomial regression was performed to determine the statistical differences between the placebo and probiotics group. SD: standard deviation; BV: bacterial vaginosis. Effect size is based on the model group differences F test.

**Table 3:** Number of days with infections during infants’ first four to six weeks of life.

	Placebo	Probiotics	*p*-Value	Effect Size	95% Confidence Interval	
	*n* = 71	*n* = 69			Lower	Upper
Days with Infections (Mean ± SD)	10.5 ± 5.57	4.7 ± 2.43	0.03	1.8	0.12	1.65

Negative binomial regression was performed to determine the statistical differences between the placebo and probiotics group. SD: standard deviation. Effect size is based on the model group differences F test.

## Data Availability

The data described in the manuscript, code book, and analytic code will be made available upon request pending application and approval. Lallemand Health Solutions Inc. is willing to share, upon receipt of a written request from qualified scientific and medical researchers, participant-level and study-level clinical trial data and protocols. To request access to this information, researchers must submit a detailed description of their intended use of the material including their objective, background, methods, statistical analysis plan, and publication plans. The submission must include a résumé of all the researchers who will access the data with a clear description of their respective roles. Data access will be granted to the anonymized study data after approval by a scientific review panel appointed by Lallemand Health Solutions Inc. and consultation from the Institutional Review Board who granted approval of the clinical trial. Our utmost concern is that participant privacy is assured. All manuscripts arising from the access of data must be provided to Lallemand Health Solutions Inc. for review at least 15 business days prior to submission to ensure conformity with our policy and participant security.

## Supplementary Materials

The following supporting information can be downloaded at: https://www.mdpi.com/article/10.3390/nu17111825/s1, Supplementary tables: Table S1. Levels of fasting blood glucose, insulin, triglycerides, and ferritin in pregnant women receiving placebo or probiotic; Table S2. Stool frequency and consistency in pregnant women receiving placebo or probiotic; Table S3. Incidence of premature rupture of membranes (PROM) in pregnant women receiving placebo or probiotic; Table S4. Participant’s weight in kilograms before and after delivery; Table S5. Post-partum depression in women receiving placebo or probiotic; Table S6. Number of infants with colic; Table S7. Frequency of reported jaundice and hyperbilirubinemia; Table S8. Proportion of daily hours of sleep in infants; Table S9. Infants’ stool frequency, consistency, and color; Table S10. Skin diseases in first year of life in infants of both groups; Table S11. Number of health problems in infants during the first year of life. Supplementary figures: Figure S1. Levels of circulating inflammatory markers and immunoglobulins in different sample types in women; Figure S2. Infants’ fecal sIgA; Figure S3. Strain recovery in mothers and infants; Figure S4. Microbiota composition in mothers’ stool samples; Figure S5. Microbiota composition in mother’s breastmilk samples; Figure S6. Toy example for the interpretation of networks analysis.
