# Supplementary material for: The Effect of Probiotics on Health in Pregnancy and Infants: A Randomized, Double-Blind, Placebo-Controlled Trial
Source: Nutrients. 2025 May 28;17(11):1825. doi: 10.3390/nu17111825 (PMC12157102; doi:10.3390/nu17111825)
Supplement: Supplementary file 1 [file nutrients-17-01825-s001.zip › nutrients-3658650-supplementary.pdf]

Supplementary Materials

Table S1. Levels of fasting blood glucose, insulin, triglycerides and ferritin in pregnant women receiving placebo or probiotic.

	Placebo (n=70)	Probiotic (n=71)	P-value
**Glucose**			
Mean ± SD	4.2 ± 0.42	4.3 ± 0.34	0.87
Minimum	4	4	
Maximum	6	5	
**Insulin**			
Mean ± SD	90.5 ± 78.93	86.5 ± 46.94	0.90
Minimum	26	11	
Maximum	596	216	
**Triglyceride**			
Mean ± SD	2.7 ± 0.96	2.6 ± 0.90	0.45
Minimum	1	1	
Maximum	6	6	
**Ferritin**			
Mean ± SD	19.9 ± 12.64	21.3 ± 13.43	0.75
Minimum	6	6	
Maximum	80	76	

Fasting blood glucose, insulin, triglyceride and iron status were measured from 70 participants in the placebo group and 71 participants in the probiotic group who provided blood samples at visit 5. To test for differences between the placebo and probiotic group, a linear model was used with treatment group as fixed effect and the individuals' baseline measure as a covariate. SD: Standard deviation.

Table S2. Stool frequency and consistency in pregnant women receiving placebo or probiotic.

	Placebo	Probiotic	P-value
**Normal weekly bowel movement – Before delivery**			
N	920	894	
Mean ± SD	5.1 ± 2.91	5.1 ± 2.84	0.89
Minimum	0	0	
Maximum	10	10	
**Normal weekly bowel movement – After delivery**			
N	349	334	
Mean ± SD	4.3 ± 2.65	4.5 ±2.83	0.29
Minimum	0	0	
Maximum	8	8	

From visit 2 to visit 7, participants were asked to complete a daily journal including the Bristol Stool Scale (BSS). They were instructed to note the consistency of each bowel movement using the BSS as a visual guide (Type 1 through 7). Slow bowel movements correspond to types 1 and 2, normal bowel movements correspond to types 3 and 4, and fast bowel movements correspond to types 5, 6 and 7. The table presents the mean number of stools rated 3 or 4 over a period of one week. To test for differences between the placebo and probiotic group over the pre/post delivery time frame, a linear mixed model was used with individuals as a random effect, and an autoregressive order 1 correlation structure. Linear contrasts were used to make comparisons of means. N: Number of bowel movements before or after delivery per group. SD: Standard deviation.

Table S3. Incidence of premature rupture of membranes (PROM) in pregnant women receiving placebo or probiotic.

	**Placebo (n=71)**	**Probiotic (n=69)**	**P-value**
Number of PROM (%)	0 (0%)	2 (2.9%)	0.24

From all the participants in both groups, only two had PROM in the probiotic group. Fisher's exact test was used to test for differences in percentages of the occurrence of PROM between groups.

Table S4. Participant's weight in kilograms before and after delivery.

	Placebo	Probiotic
**Prior to pregnancy**		
N	87	84
Mean ± SD	68.1 ± 14.75	69.9 ± 13.97
Minimum	44	45
Maximum	113	116
**Visit 2**		
N	90	90
Mean ± SD	78.1 ± 15.21	78.3 ± 13.50
Minimum	54	51
Maximum	130	113
**Visit 5**		
N	73	70
Mean ± SD	82.2 ± 15.45	82.5 ± 14.57
Minimum	57	54
Maximum	140	147
**Visit 7**		
N	69	69
Mean ± SD	74.3 ± 18.90	73.0 ± 13.26
Minimum	49	46
Maximum	168	108

Participants' weight was measured at visits 2, 5 and 7. At visit 2, participants were also asked to recall their weight prior to pregnancy. With an allowable difference of 1.45 kg, the placebo and the probiotic groups are equivalent. Equivalence was tested using the TOST procedure on the log scale to meet the assumptions of the model. To test for differences between the placebo and probiotic group at each visit, the change from baseline, and difference in change between groups, a linear mixed model was used with treatment group, visit (visits 2, 5 and 7), and their interaction as fixed effects, individual as a random effect, and an autoregressive order 1 correlation structure. Linear contrasts were used to make comparisons of means. The response measures were log transformed to meet the assumptions of the model, and all least square means were back transformed using the delta method.

Table S5. Post-partum depression in women receiving placebo or probiotic.

	Placebo	Probiotic	P-value
**Visit 2**			
N	87	85	
Mean ± SD	5.3 ± 4.20	4.8 ± 3.76	0.57
Minimum	0	0	
Maximum	18	20	
**Visit 7**			
N	70	69	
Mean ± SD	4.1 ± 3.05	4.8 ± 3.30	0.06
Minimum	0	0	
Maximum	14	13	

Postpartum depression was assessed at visit 7. Participants were instructed to complete the 10-question Edinburgh Postnatal Depression Scale (EPDS). Mothers who score above 13 are likely to be suffering from depression of varying severity. The scale asks mothers to rate how they have felt during the previous week. To test for differences between the placebo and probiotic group, a linear model was used with treatment group as fixed effect and the individual's baseline (visit 2) measure as a covariate. The response was square transformed, and least square means were back transformed using the delta method.

Table S6. Number of infants with colic.

	Placebo (n=71)	Probiotic (n=69)	P-value
Infants with colic (%)	5 (7%)	6 (8.6 %)	1

Participants reported daily from date of delivery until visit 7 the status of the baby over the last 24 hours (sleeping, awake and crying, unsoothable crying, or cannot remember). Colic is defined as unsoothable crying for at least three hours for one day, which is repeated for at least three days within two weeks. Statistical difference between groups was tested using Fisher exact test.

Table S7. Frequency of reported jaundice and hyperbilirubinemia.

	**Placebo (n=71)**	**Probiotic (n=69)**	**P-value**
Infants with jaundice (%)	10 (14.1 %)	20 (29 %)	0.04
Infants with hyperbilirubinemia	0	0	1

Probable jaundice was recorded at visit 7 based on the mothers' report of their child's color. Fisher exact test was performed to determine the statistical difference between groups. Hyperbilirubinemia (diagnosed jaundiced) was also recorded at visit 7. No infant had hyperbilirubinemia in either group.

Table S8. Proportion of daily hours of sleep in infants.

	Placebo	Probiotic	P-value
N	1844	1551	0.18
Mean ± SD	0.6 ± 0.15	0.6 ± 0.16	
Minimum	0	0	
Maximum	1	1	

Sleeping time was calculated considering the mother's report of what her baby was doing during the day; sleeping, crying or awake. To compensate for the fact that not all mothers reported the baby's activities/sleep pattern over a full 24h-period, we tested for differences in percentages of sleeping time using a generalized linear model with binomial distribution. Linear contrasts were used to make comparisons of means. Least square means were back transformed using the delta method. It was important to compare the ratio hours sleep/total hours reported between groups, it is 0.6 in the placebo group and the probiotic group, the difference is not significant between groups ($p = 0.18$).

Table S9. Infant's stool frequency, consistency, and colour.

	Weekly stools		Proportion of soft stools in a week		Proportion of yellow stools in a week	
	**Placebo**	**Probiotic**	**Placebo**	**Probiotic**	**Placebo**	**Probiotic**
**N**	337	329	67	66	67	66
Mean ± SD	29.1 ± 13.80	26.3 ± 13.23	0.5 ± 0.38	0.5 ± 0.36	0.3 ± 0.34	0.4 ± 0.38
Minimum	1	0	0	0	0	0
Maximum	63	60	1	1	1	1

Participants were instructed to note daily the number of bowel movements their infant had between birth and 7. Participants also noted the colour and consistency of their infant's stool using the Amsterdam Infant Stool Scale (AISS) as a guide. The AISS breaks down consistency into 4 categories (Type A: watery, Type B: soft, Type C: formed, and Type D: hard) and colour into 6 categories (Type I: yellow, Type II: orange, Type III: green, Type IV: brown, Type V: dark green, and Type VI: grey). To test for differences between the placebo and probiotic group for number of infant stools in a week, a linear mixed model was used with individuals as a random effect, and an autoregressive order 1 correlation structure. The response measures were square root transformed to meet the assumptions of the model and linear contrasts were used to make comparisons of means. To test for differences in proportions a generalized linear model with binomial distribution was used (same model as above). Linear contrasts were used to make comparisons of means. All least square means were back transformed using the delta method. There are no significant differences between the placebo and probiotic groups in mean weekly infant stool number ($p = 0.16$), consistency ($p = 0.76$), or colour ($p = 0.55$).

Table S10. Skin diseases in first year of life in infants of both groups.

	Placebo (n=67)	Probiotic (n=66)	P-value
Number of skin diseases (%)	6 (9%)	14 (21.2 %)	0.06

Skin diseases (dermatitis, eczema, and rash) occurring in the first year of life (i.e. between the last study visit [visit 7] at 4-6 weeks of life and the follow-up phone call [visit 8] after one year) were reported by mothers. They indicated if the infant had been diagnosed with any skin diseases since birth. Fisher exact test was performed to determine the statistical difference between groups.

Table S11. Number of health problems in infants during the first year of life.

	Placebo (n=67)	Probiotic (n=66)	P-value
Mean ± SD	0.8 ± 1.16	0.8 ± 1.08	0.62
Minimum	0	0	
Maximum	4	4	

Overall health status (e.g., concerns with crying, feeding, asthma, allergies, developmental delays) in the first year of life were reported by the mothers at the follow-up phone call (visit 8). Mothers indicated if the infant had any health issues since birth. Overall health status of infants was similar between groups, $p = 0.62$, Wilcoxon rank sum test.

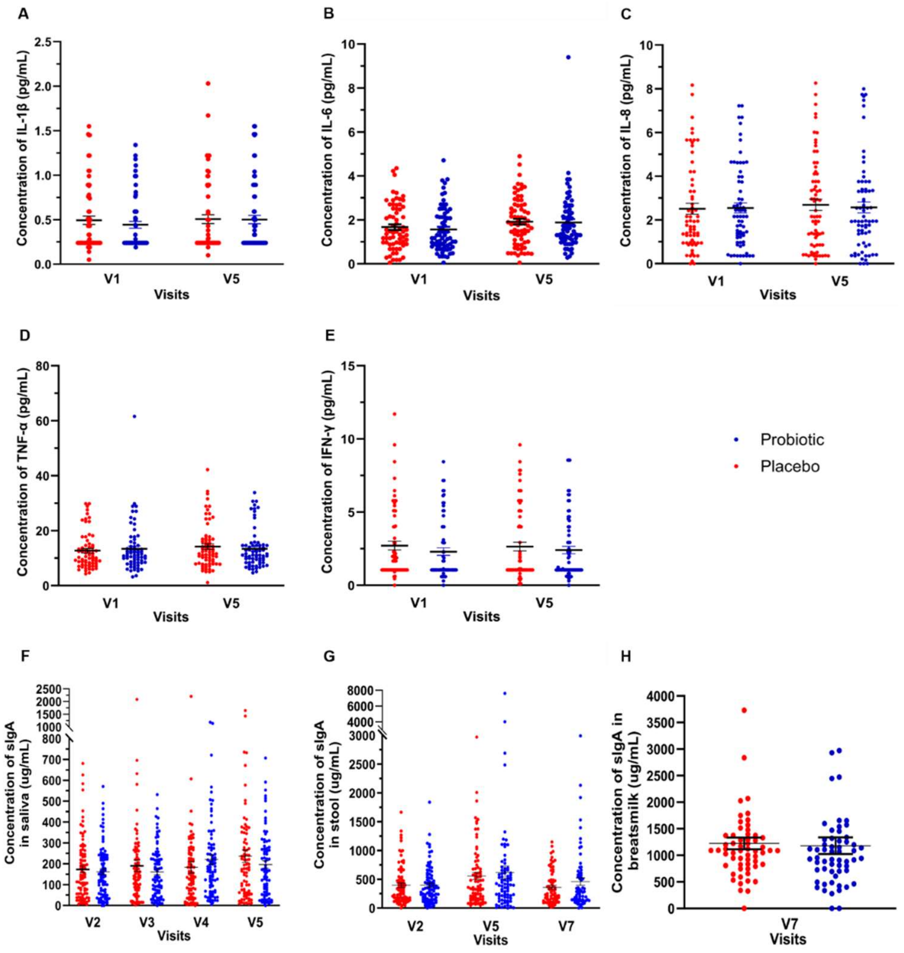

Figure S1. Levels of circulating inflammatory markers and immunoglobulins in different sample types in women.

(A) Il-1β (V1: p=0.59; V5: p=0.67); **(B)** IL-6 (V1: p=0.41; V5: p=0.51); **(C)** IL-8 (V1: p=0.70; V5: p=0.77); **(D)** TNF-α (V1: p=0.71; V5: p=0.61) and **(E)** INF-γ (V1: p= 0.22; V5: p=0.68). Wilcoxon rank sum test was performed to determine significant difference between the placebo and probiotic groups. At V1, n=67 and 69 for the placebo and probiotic group, respectively. At V5, n=70 for both groups. **(F), (G)** and **(H)** show the distribution of sIgA in saliva, stool and breastmilk, respectively, before and/or after delivery. A Wilcoxon rank sum test was performed to determine significant difference between the placebo and probiotic groups. **(F)** For salivary samples collected before delivery and at various visits, the numbers and p-values for the placebo and probiotic groups were as follows: at V2, n=88 (placebo) and n=87 (probiotic) with p=0.98; at V3, n=86 (placebo) and n=82 (probiotic) with p=0.76; at V4, n=84 (placebo) and n=83 (probiotic) with p=0.14; and at V5, n=80 (placebo) and n=78 (probiotic) with p=0.72. **(G)** For stool samples collected before and after delivery, the numbers and p-values for the placebo and probiotic groups were as follows: at V2, n=87 (placebo) and n=84 (probiotic) with p=0.89; at V5, n=80 (placebo) and n=79 (probiotic) with p=0.87; and at V7, n=69 for both groups with p=0.63. **(H)** For breastmilk samples collected after delivery at V7, the numbers and p-values were as follows: n=59 for the placebo group and n=58 for the probiotic group with p=0.22. All data in these graphs represents mean ± SD. sIgA: Secretory Immunoglobulin A; IL: Interleukin; INF: Interferon; TNF: Tumor Necrosis Factor; V: Visit.

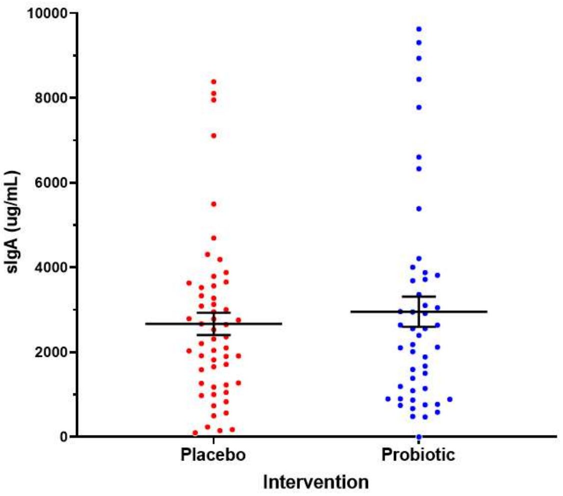

Figure S2. Infants' fecal sIgA.

Infant stool samples were provided at visit 7. sIgA levels were measured from 54 participants in the placebo group and 47 participants in the probiotic group. The mean fecal sIgA levels are 2668.2 ug/mL in the placebo and 3029.3 ug/mL in the probiotic group. The difference between the groups is not significant based on the Wilcoxon rank sum test (p=0.80).

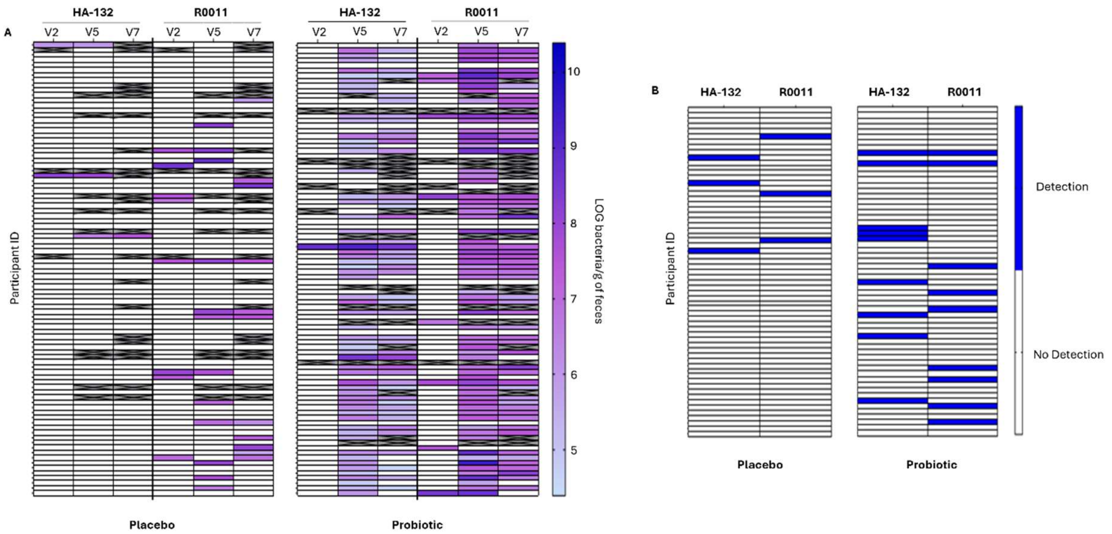

Figure S3. Strain recovery in mothers and infants.

HA-132 and R0011 detections in mothers' stool samples **(A)** and infants' stool samples **(B)** performed by Real-Time qPCR. Participants provided stool samples at visits 2, 5 and 7. The infant stool samples were provided at visit 7. HA-132: *B. bifidum*; R0011: *L. rhamnosus*; V: Visit.

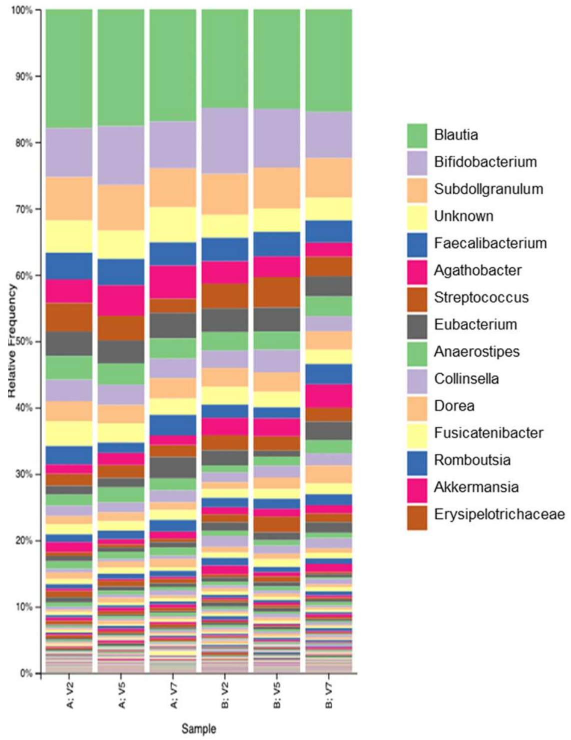

Figure S4. Microbiota composition in mothers' stool samples.

Gut microbiome composition at genus level of participants in both groups at V2, V5 and V7. The three bars on the left are for group A (Placebo) and the three on the right are for group B (Probiotic). For each group, the three visits (V2, V5 and V7) are shown left to right.

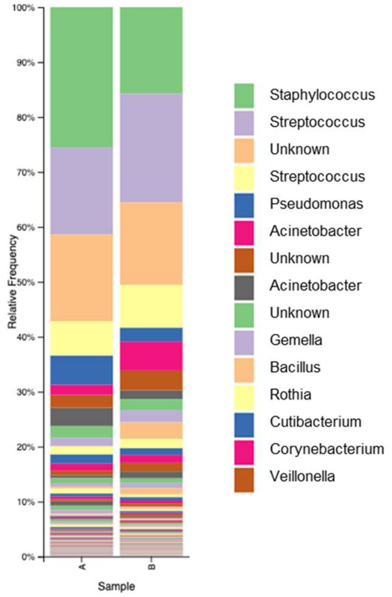

Figure S5. Microbiota composition in mother’s breastmilk samples.

Breastmilk microbiome composition at genus level of participants in group A (Placebo) and group B (Probiotic) at visit 7.

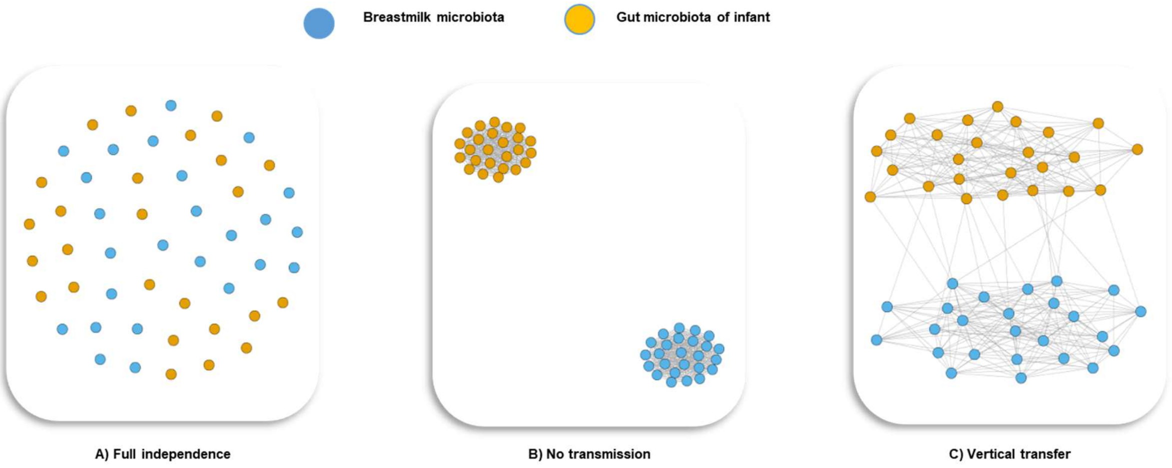

Figure S6. Toy example for the interpretation of networks analysis.

(A) An example of full independence between and within breastmilk microbiota and infant's gut microbiota. **(B)** An example of no transmission between breastmilk microbiota and infant's gut microbiota. **(C)** An example of vertical transfer between breastmilk microbiota and infant's gut microbiota.
